# Bringing the clinical laboratory into the strategy to advance diagnostic excellence

**DOI:** 10.1515/dx-2020-0119

**Published:** 2021-01-06

**Authors:** Ira M. Lubin, J. Rex Astles, Shahram Shahangian, Bereneice Madison, Ritchard Parry, Robert L. Schmidt, Matthew L. Rubinstein

**Affiliations:** Division of Laboratory Systems, Centers for Disease Control and Prevention, 1600 Clifton Rd., NE Mail Stop V24-3, GA 30329, Atlanta, GA, USA; Division of Laboratory Systems, Centers for Disease Control and Prevention, Atlanta, GA, USA; Department of Pathology, University of Utah School of Medicine, Salt Lake City, UT, USA; Department of Clinical Laboratory and Medical Imaging Sciences, Rutgers, The State University of New Jersey, Newark, NJ, USA

**Keywords:** diagnostic error, diagnostic excellence, laboratory practice, quality

## Abstract

**Objectives:**

Clinical laboratory testing provides essential data for making medical diagnoses. Generating accurate and timely test results clearly communicated to the treating clinician, and ultimately the patient, is a critical component that supports diagnostic excellence. On the other hand, failure to achieve this can lead to diagnostic errors that manifest in missed, delayed and wrong diagnoses.

**Content:**

Innovations that support diagnostic excellence address: 1) test utilization, 2) leveraging clinical and laboratory data, 3) promoting the use of credible information resources, 4) enhancing communication among laboratory professionals, health care providers and the patient, and 5) advancing the use of diagnostic management teams. Integrating evidence-based laboratory and patient-care quality management approaches may provide a strategy to support diagnostic excellence. Professional societies, government agencies, and healthcare systems are actively engaged in efforts to advance diagnostic excellence. Leveraging clinical laboratory capabilities within a healthcare system can measurably improve the diagnostic process and reduce diagnostic errors.

**Summary:**

An expanded quality management approach that builds on existing processes and measures can promote diagnostic excellence and provide a pathway to transition innovative concepts to practice.

**Outlook:**

There are increasing opportunities for clinical laboratory professionals and organizations to be part of a strategy to improve diagnoses.

## Introduction

Medical errors are estimated as the third leading cause of death in the United States with up to one third of these associated with diagnostic errors [1, 2]. A 2014 study by Singh et al. derived estimates from large observational studies of the US population to determine that one in 20 adults is affected by a diagnostic error during their lifetime [3]. In 2015, the National Academy of Medicine (NAM), formally the Institute of Medicine, published a report, *Improving Diagnosis in Health Care*, highlighting the critical need to understand and address shortcomings in the diagnostic process [4]. The report recognizes that only through an integrative systems approach across medical disciplines and with the inclusion of patient input can healthcare organizations measurably reduce diagnostic errors. This approach includes clinical laboratory practice as integral to the diagnostic process [5]. This narrative review will summarize current initiatives and future prospects for improving diagnoses that include reducing diagnostic error, emphasizing the role of the clinical laboratory.

## The intersection of diagnostic excellence and clinical laboratory practice

**Figure 1:**
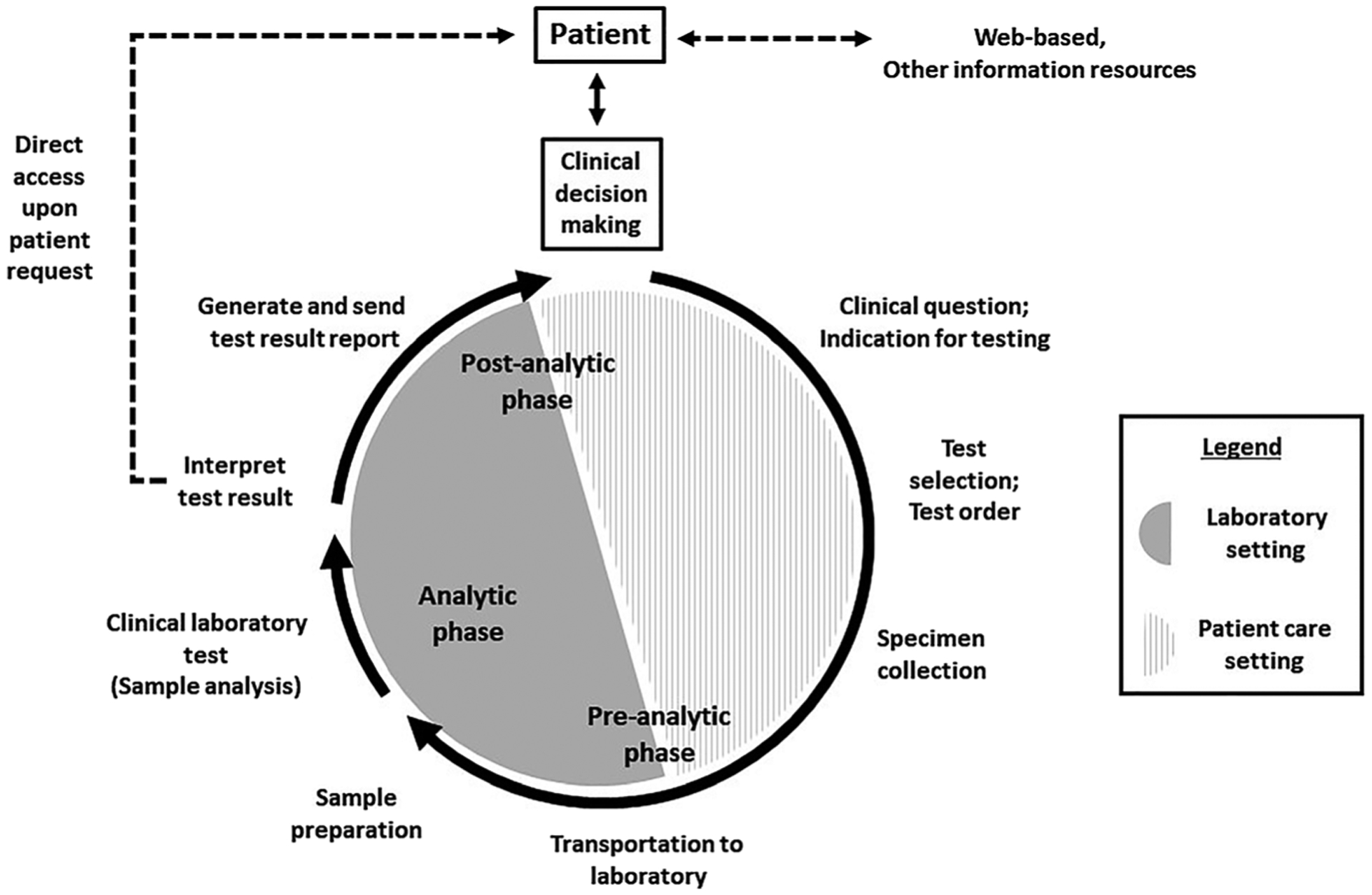
The total testing process describes the lifecycle of a clinical laboratory test [6, 7]. The process is divided into the pre-analytic, analytic, and post-analytic phases of testing. The pre-analytic phase includes all steps that occur prior to perforrning the clinical test. The analytic phase includes performance of test procedures, quality control, calibration, and verification procedures, and documentation of testing data. The post-analytic phase includes all steps that occur after the clinical test is performed. The pre- and post- analytic phases of testing include steps that occur in both the laboratory and patient-care settings. Dotted lines indicate aspects more recently entering practice that provide patients direct access to test results and access to web-based and resources that contain information about clinical laboratory testing.

For the purpose of this manuscript, we define diagnostic excellence as a systems-level state that effectively integrates health care knowledge, skills, and resources for continuous and measurable improvement of diagnoses, and reduction of risk or occurrence of diagnostic errors, while continuing to meet overall needs of patients and health systems. The intersection of diagnostic excellence and laboratory practice can be traced to the conceptual development of the total testing process. Lundberg first described the “life cycle” of a clinical laboratory test in 1981, which was subsequently defined as the “total testing process” (TTP) by the US Centers for Disease Control and Prevention (CDC) in 1986 and revisited in 2011 [6–8]. (Figure 1) Lundberg emphasized the need for continuous assessment to support the added value of laboratory testing by stating “clinicians and laboratory professionals should all be concerned about the effects of the laboratory test and whether its performance was useful for the patient or public’s health” [9]. Building on these earlier efforts, a 2008 CDC report, *Laboratory Medicine, A National Status Report*, advocated that the clinical laboratory has value beyond test performance through enhanced engagement with other health care providers to improve health outcomes [10]. At the time, there were a limited number of studies that linked elements of the total testing process to accurate and timely diagnoses in the patient setting, with far less attention to linkages with measurable health outcomes. In 2013, Epner et al. revisited this concept by advocating for an outcomes-based approach for laboratory medicine that links laboratory processes to accurate and timely diagnoses to support diagnostic excellence [11].

Antimicrobial stewardship provides an illustrative example of practices that support diagnostic excellence [12–14]. Antimicrobial stewardship is a system-based, multidisciplinary approach designed to facilitate the timely administration of the optimal antibiotic to a patient diagnosed with an infectious disease that fundamentally relies on accurate and timely test results available to inform clinical decision making [13, 15–18]. Properly implemented, antimicrobial stewardship includes active engagement of the treating physician, infectious disease physician, laboratory professional(s), pharmacists, and other medical professionals.

Antimicrobial stewardship is closely linked to making an accurate and timely diagnosis, sometimes referred to as “diagnostic stewardship”. The practice of testing for blood culture contamination to differentiate between blood stream infection and an external contamination event (e.g., non-sterile tube stoppers) provides as an example that links microbial/diagnostic stewardship to diagnostic excellence [16]. Blood culture contamination can occur at various stages of the testing process, from sample collection to sample analysis. Failure to recognize blood culture contamination can result in misdiagnosis, inappropriate antibiotic use, and extended hospital stays [19]. It is estimated that in the US, blood culture contamination rates range from 0.6 to 12.5% with the highest rates occurring in emergency department settings [20]. Professional recommendations state that blood culture contamination should not exceed 3% [21, 22]. Most notable is the importance of detecting blood culture contamination when evaluating a patient for septicemia, a leading cause of hospital deaths in the US [23]. Accurate and timely diagnosis of septicemia can prolong survival consistent with the concept of diagnostic excellence [24].

## Linking the total testing process to making a clinical diagnosis

The TTP is often described in terms of the pre-analytic, analytic, and post-analytic phases of testing [10]. While the laboratory typically has rigorous practices in place to monitor the analytic phase of the test, less control and monitoring are afforded to the pre- and post-analytic phases of testing, where the majority of errors were found [25, 26]. The pre- and post-analytic phases of testing occur in both clinical and laboratory settings. These phases include test selection/ordering and result interpretation/reporting. Errors across the TTP that compromise diagnoses were reviewed by Epner et al. and described as “testing-related diagnostic errors” [11].

Efforts to quantitate diagnostic errors linked to the TTP are primarily available through medical liability claims and voluntary reporting from patient safety organizations. A study by Coverys, a medical liability insurance and risk management service organization, examined 3,466 claims across medical practice settings from 2013 through 2017 and reported that 52% of claims and 55% of indemnity paid were associated with steps in the TTP that linked to diagnostic errors [27]. A 2014 Emergency Care Research Institute (ECRI) report summarized 420 test-related diagnostic errors across medical settings from 2011 through 2013. Descriptions of these errors were submitted by ECRI’s patient safety and other member organizations [28]. For the errors identified, 74% occurred in the pre-analytic, 4% in the analytic, and 22% in the post-analytic phases of testing. The report showed that test-related issues associated with missed, wrong, or delayed diagnoses were, at least in part, attributable to absent or incomplete specimen labeling, poor specimen quality, ordered test not performed, or missing, delayed or wrong results. A second ECRI report was published that analyzed 4000 patient safety events collected during 2017–2018, of which 035 involved diagnostic testing that included laboratory analyses, imaging pathology and other diagnostic testing procedures. There were 1,408 laboratory errors that contributed to 69% of all diagnostic errors [29]. Most errors were associated with test ordering, specimen collection, and results reporting. Another study looked at unexpected return visits for medical follow up in a large urban Veterans Affairs Medical Center and a large integrated private health care system and found 57% of diagnostic errors in this cohort were associated with breakdowns in the ordering of follow up clinical tests (a component of the post-analytic process of the TTP) [30]. This and other findings correlated with unplanned rehospitalization or emergency department/urgent care visits. These findings collectively suggest opportunities for the laboratory to engage in quality improvement initiatives collaboratively with their patient care colleagues to measurably reduce diagnostic errors.

## Examples of innovations that address vulnerabilities in the total testing process

The following examples highlight a selection of initiatives that laboratories are taking or can take to reduce or prevent diagnostic errors, or otherwise promote diagnostic excellence with respect to vulnerable steps of the TTP. Areas of focus include improving test utilization, leveraging clinical and laboratory data derived from the electronic health record to improve diagnoses, promoting effective use of credible information resources, improving communication among laboratory professionals, other health care providers, and patients, and using a diagnostic management team (DMT) approach to support diagnostic excellence [31].

## Improving test utilization

Diagnostic error can result from inappropriate test utilization (e.g., mis-, under-, or over-utilization) where practices are not consistent with current subject-specific expert knowledge or evidence-based standards for usage and cost-effectiveness [32]. Zhi et al. reported that test over-utilization can be significant and vary by clinical setting, test volume, and measurement criteria [33]. This report also noted a sparsity of studies describing the under-utilization of clinical laboratory testing. Systematic reviews and meta-analyses published from 2012 to 2018 identified practices that support appropriate laboratory test utilization [32, 34]. These include the use of modified computerized physician order entry (CPOE) that provides alerts for redundant tests ordered within a specified time interval, display of test cost at the time of ordering, a limit of test availability provided in the CPOE user interface, and reflex testing. A few studies extended these findings to aspects relevant to achieving diagnostic excellence in proposing the use of “demand management” where multiple modalities are implemented to promote appropriate test utilization [35, 36]. These modalities include use of laboratory diagnostic algorithms, educational interventions, gate keeping strategies, and review of tests offered. While evidence supports these practices, broad implementation and evaluation across laboratory and clinical settings has yet to occur.

Another example looks at an initiative designed to review test orders to determine whether the test(s) requested is appropriate to the indication for testing stated on the test requisition. A 2014 publication reported a reduction in unnecessary testing by a reference laboratory, achieved through follow-up with the ordering clinician following laboratory-based genetic counselor review of genetic test orders [37]. Findings showed that 26% of test requests for complex genetic tests were changed as a result of this process. This achieved an average reduction in billing of $48,000 per month. These findings equated with a reduction in unnecessary testing and allude to a reduction in wrong, delayed- and misdiagnoses, although these latter outcomes were not measured. The extent to which inappropriate test orders over the range of indications for testing contribute to wrong, missed, or delayed diagnoses is not known, but may be significant based on these findings. This suggests an opportunity to further explore a role for the laboratory in working with clinicians across medical disciplines to assure appropriate and cost-effective test utilization.

## Leveraging clinical and laboratory data derived from the electronic health record to improve diagnoses

The evolution of health information technology (IT) systems is providing essential tools to improve test utilization and demonstrate measurable improvements to making accurate and timely diagnoses. For example, SureNet, a program developed within the Southern California Kaiser Permanente healthcare system, is an innovative program that uses a tracking and alert system based on clinical and laboratory data abstracted from patient electronic records [38]. This program was initially created to address post-analytic errors associated with failure to follow up within a 90-day time-frame with patients having a low estimated glomerular filtration rate based on an elevated creatinine test result. The initial study used a retrospective cohort of 12,396 individuals from which 6,981 were contacted and had follow-up testing, and 1,550 were diagnosed with chronic kidney disease. In the absence of follow up testing facilitated by SureNet, diagnoses of chronic kidney diseases would have been missed or delayed [39]. This process transitioned to standard of care and since this study, 54 conditions are now tracked within the SureNet program (https://permanente.org/reducing-diagnostic-errors/, accessed November 4, 2020). This is one of the few examples that was both evidence-based and implemented to sustainably collect and analyze data that link clinical laboratory testing to a identify patients at risk for a debilitating disease where early diagnosis has significant value to reducing morbidity and mortality. A similar approach was taken to diagnose patients at risk for early organ dysfunction who were prescribed disease-modifying anti-rheumatic drugs (DMARDs) [40]. This study leveraged abstracted electronic health record (EHR) data contained within the local medical data warehouse to identify patients not receiving guideline compliant testing to detect potential DMARD-related organ toxicities. Laboratory professionals led this quality improvement initiative, taking on several roles that included data analysis, in collaboration with Kaiser’s data management team. An automated interactive voice response system was developed to contact out-of-compliance patients and communicate the need for testing. The short-term pilot was deemed successful in bringing ~10% of patients back into testing compliance. This finding supported the decision to transition this process to standard of care. Now implemented within the practice case setting, it will be helpful to determine whether additional levels of guideline compliance are achieved and whether this leads to decreasing the number of patients exhibiting organ toxicity attributable to DMARDs.

Clinical laboratory professionals continue to explore ways to improve the TTP, taking advantage of assets that may not have been readily accessible, such as using data abstracted from patients’ electronic health records for novel applications. For example, to reduce false positive test results indicative of the presence of certain drugs in urine, data from electronic health records corresponding to nearly 700,000 urine drug screens across 10 assays were used to assess patients’ previous medication exposures [41]. Results from this study identified cross-reactive substances that interfered with laboratory testing that otherwise would likely be missed. Findings from these studies led to improvements in the test method and a reduction in false-positive drug results relevant to making accurate and timely diagnoses. Laboratories are also innovating solutions to use patient data to modify standard reference ranges used to inform rule in and rule out differential diagnoses [42]. Patients who are under treatment may have chronically abnormal results when reference intervals derived from healthy individuals are applied. To reduce the information overload that results by frequently alerting clinicians of out-of-range laboratory test results that may in fact be within the normal range for inpatient populations, some laboratories have modified their reference ranges using test values often seen in hospital settings. Compared to traditional alert levels based on apparently healthy subjects, such customized alerts led to decreased sensitivity (77 vs. 85%) and negative predictive value (97.1 vs. 98.6%), but significantly (p<0.001) higher specificity (79 vs. 61%) and positive predictive value (28 vs. 11%) for calling a laboratory result abnormal. The report detailing this study also considers additional steps needed to transition the process described to standard of care in hospital settings. This includes looking at larger inpatient populations to better understand the risks, harms, and clinical performance that influence the diagnostic process.

## Promoting effective use of credible information resources and communication among laboratory professionals, other health care providers, and patients

Clinical laboratory tests are increasing in number and complexity, necessitating an understanding of their uses and limitations to support accurate and timely diagnoses. This requires specialized knowledge that is within the domain of clinical laboratory professionals. From a clinician perspective, test selection and ordering (pre-analytic), interpretation and application of the test result to clinical decision making (post-analytic) require knowledge of the uses and limitations of available tests. Maintaining adequate knowledge is challenging considering the rapid evolution, complexity and increasing number of tests available to clinicians [43, 44]. One study suggests that physicians primarily rely on knowledge achieved during their initial medical training and the advice of colleagues, who may or may not be fully informed of available testing [45]. To address this knowledge gap, there are an increasing number of resources to better inform an understanding of available tests. For example, mobile apps that provide current recommendations for diagnostic evaluations that include recommended testing modalities have gained broad acceptance among the clinician community [46]. In addition, there is significant work toward evolving the integration and use of clinical decision support tools within the EHR to expedite the diagnostic evaluation and clinical management of the patient [47, 48]. The extent to which these innovations improve diagnoses across medical disciplines is not established and requires further study [49]. Availability of and access to information about testing is helpful but in some cases may not be sufficient to mitigate test-related diagnostic errors because they do not address cognitive issues that can compromise the diagnostic process [11].

From a patient perspective, clearly communicated test results that include information about the test’s uses and limitations are important for making informed health care decisions. Patients and the public in general, have access to test-related information beyond what is shared by their health care provider. Patient portals, available in both independent medical practices and larger institutions, provide the opportunity for access to abstracted information contained within the patient’s electronic health record that includes test-related information [50]. In the US, patients can access test results directly from the laboratory, a consequence of a 2014 federal rule designed to empower patient decision making outside the context of clinician-provided information [51]. In addition, there is significant information available about clinical laboratory testing and its application to the diagnostic evaluation and other aspects of healthcare on the Internet [52]. Studies suggest that collectively these information resources can be of value especially when findings are discussed with one’s healthcare provider [49, 52]. For example, establishing one’s family history using an online tool can be helpful to identify persons at-risk for breast cancer and expedite the clinical evaluation (https://bcrisktool.cancer.gov/calculator.html, accessed November 4, 2020). Conversely, the credibility of test-related and other healthcare information available on the Internet varies and this can potentially compromise accurate and timely diagnoses depending how this information is understood and used by the patient to inform decisions [53, 54].

## Use of the diagnostic management team approach to support diagnostic excellence

A growing number of diagnostic evaluations are complex and require specialized medical knowledge across medical disciplines. This complexity can translate to multiple medical referrals where diagnoses are made and vary among physicians. This can compromise establishing an accurate and timely diagnosis [55]. One approach to address challenges associated with the medical referral model is the use of DMTs [56]. The DMT approach leverages a multi-disciplinary health care team, which includes the patient and medical professionals that works collaboratively to make team-based decisions with the intent to derive at an accurate and timely diagnosis. For example, the DMT approach has been useful in the timely and accurate diagnosis of active infections, permitting judicious administration of antibiotics [15, 17]. For this example, The DMT team can include clinicians, laboratory professionals, pharmacists and infectious disease specialists, among others. DMTs can reside in and be led from patient care or laboratory settings. Verna et al. present a case for a DMT based out of the laboratory to assure that the uses and limitations of clinical laboratory testing inform appropriate test ordering and result interpretation [57]. Although the utility of DMTs appears self-evident, studies are lacking that show use of these multi-disciplinary teams consistently improves accurate and timely diagnosis that is effectively communicated to the patient across practice settings [58]. Such evidence is important to support the business case for diagnostic teams since there is a cost to their development, coordination, and integration into the patient care continuum.

## An advanced degree offering in clinical laboratory science that supports the changing healthcare environment

Another innovation is the development of Doctor of Clinical Laboratory Science (DCLS) programs. These programs are designed to develop professionals to support the laboratory to meet demands as the health care system continues to evolve. These degrees are inherently multi-disciplinary, rather than focused on a single traditional laboratory discipline. The need for these professionals was formally envisioned at a conference in 2000 hosted by the National Accrediting Agency for Clinical Laboratory Sciences with programmatic elements collaboratively developed over the next decade with the involvement of several professional organizations [59]. As of 2020, DCLS programs are now in place at a few academic institutions to produce graduates trained to advance quality laboratory practices, facilitate collaboration among healthcare disciplines, translate research findings to practice, and integrate laboratory functions into broader aspects of healthcare delivery. As of 2020, these programs do not contribute to eligibility for Board exams or other certifications recognized by federal or state agencies. In time, this may change if it is found that DCLS graduates fill gaps in essential expertise and contribute to cost-effective operational success as laboratories assume expanded roles.

## Quality measures and quality management: key to monitoring and supporting diagnostic excellence

Advancing diagnostic excellence in a meaningful way requires measurement. For example, Medical Quality Indicators developed by the International Federation for Clinical Chemistry Working Group on “Laboratory Errors and Patient Safety” (IFCC WG-LEPS) spans key laboratory processes, laboratory support processes, and laboratory outcomes; while full validation of these indicators is ongoing, and can be expected to evolve over time, they represent an essential contribution to laboratories seeking to systematically prioritize improvement actions in view of patient safety and ISO 15189’s robust quality management perspective [60, 61]. Several professional and governmental bodies developed and/or endorsed quality indicators applicable to clinical and laboratory processes (Table 1). Quality indicators in laboratory and patient care practice are used to measure concordance with acceptable practices and as measures for meeting specified criteria. These metrics can also be used to benchmark practices and compare outcomes across one or more organizations. Examples can be cited. The Model of Quality Indicators developed under the IFCC WG-LEPS, noted above, is designed to support a proactive system for defining quality indicators and monitoring performance that in turn is directed to decreasing the error rate associated with the total testing process [60]. Similarly, The College of American Pathologists sponsors Q-Probes and Q-Tracks that represent, respectively, short- and long-term assessment of key processes to aid in quality improvement initiatives [62].

As of 2020, only a few quality indicators link laboratory testing and processes to clinical diagnoses. For example, a Healthcare Effectiveness Data and Information Set measure was developed to assess accurate screening methods for high-risk human papillomavirus testing relevant to the timely diagnosis of cervical cancer in the patient care setting (https://www.ncqa.org/hedis/measures/, accessed November 4, 2020). Advancing diagnostic excellence will rely on quality indicators that connect elements of the TTP to the diagnostic evaluation that occurs in the patient care setting. One promising model to build upon, and which is amenable to an enhanced laboratory component, is the framework described within the NAM report, *To Err is Hum*an, *Building a Safer Health System*, which details quality domains that include patient safety, effectiveness, equity, patient-centeredness, timeliness, and efficiency [63]. In 2009, Shahangian and Snyder built upon this framework by cross-walking categories of laboratory quality indicators by phase of the TTP to these NAM quality domains [64]. A similar and expanded approach was published by the National Quality Forum in 2019 that reported a measurement framework to assist in reducing diagnostic harm, applicable to both laboratory and patient-care settings (http://www.qualityforum.org/Publications/2019/10/Reducing_Diagnostic_Error_-_Measurement_Considerations.aspx, accessed November 4, 2020). This report details 17 new measures associated with diagnostic efficiency and accuracy sub-domains of the diagnostic process and outcome domains. Another model informed from the earlier NAM efforts mentioned above is the Safer DX Framework that describes a process to integrate elements of the diagnostic process with metrics constructed to collect data that can inform policy and practice in support of health care value [65]. While this framework is patient care focused, issues of testing and laboratory practice are recognized as important to the diagnostic evaluation.

Transitioning these models to practice requires engagement of professional organizations, practitioners, and governmental agencies. As of 2020, local and national infrastructure for advancing diagnostic excellence that includes a strong laboratory component is limited. Questions requiring additional exploration in developing such an infrastructure include:

1. What changes are needed relevant to the current practice culture and the traditional laboratory-centric quality management system approach?
2. What quality management approaches and indicators are useful to both laboratories and healthcare systems to meet criteria associated with diagnostic excellence and inform quality improvement initiatives?
3. What data are useful to collect, analyze, and share to systematically promote quality management of the TTP to promote diagnostic excellence in the laboratory and patient care settings?

Professional organizations and governmental entities are studying and considering strategies to advance diagnostic excellence. A few of these efforts are described in Table 2. The majority of these emphasize either the patient care or laboratory setting with a few taking a more integrative approach. Several common themes are derived from these evolving efforts that include the importance for:

Developing and applying a strong data-driven evidence base to describing and advancing diagnostic excellence
Accounting for patient involvement in the diagnostic process
Continuous quality improvement that includes mechanisms for feedback to laboratory and clinical professionals about best practices for achieving accurate and timely clinical diagnoses
Developing and using multi-disciplinary healthcare teams (including DMTs) that include laboratory professionals to assure accurate, timely, and effective communication of diagnoses and assure the overall quality of patient care [66].

In 2020, the Agency for Health Care Research and Quality (AHRQ) published a brief that addressed the importance of measuring diagnostic process performance as essential to engaging in efforts to reduce diagnostic errors and improve patient safety [67]. AHRQ recognized the absence of a coordinated US strategy to measure diagnostic safety and focused their comments on implementing measurement strategies at the level of the healthcare organization. Building upon this report, an opportunity emerges to develop regional and national strategies for diagnostic surveillance that supports data collection across healthcare organizations that in turn can inform studies and quality improvement initiatives to advance diagnostic excellence [68]. A system for diagnostic surveillance can build on existing efforts designed to collect and analyze data on medical errors, such as the ECRI work described earlier. In fact, voluntary reporting of medical errors was a topic first critically addressed in the NAM report, *To Err is Human: Building a Safer Health System* [63]. This work supports the concept for an expanded quality management system approach that encompasses both clinical and laboratory settings as opposed to current systems that are less integrated. The quality management system approach can provide a framework for continuous quality improvement across laboratory and patient care settings. An integrated quality management approach may evolve from established systems that include the Clinical Laboratory Improvement Amendments (CLIA) quality system standards, CLSI quality systems essentials, total quality management, Six Sigma, ISO 15189, and others. (https://www.who.int/ihr/publications/lqms_en.pdf, accessed November 4, 2020) [61, 69–72]. These systems systematically monitor performance to reduce, identify, and mitigate errors and promote high quality practices.

## Summary

### Opportunities to bring the laboratory into a strategy to advance diagnostic excellence

The NAM report, *Improving Diagnosis in Healthcare*, described several goals considered essential for improving diagnoses and reducing diagnostic errors [4]. The concept of diagnostic surveillance and quality management across laboratory and patient care settings intersects with several goals outlined in the NAM report that propose to develop and deploy approaches to identify, learn from, and reduce diagnostic errors and near misses in clinical practice, and establish a work system and culture that supports the diagnostic process and improvements in diagnostic performance. For example, one approach may be to expand the concept of diagnostic stewardship, introduced earlier in this manuscript, beyond the infectious disease realm to be inclusive of other laboratory disciplines across a range of medical disciplines [16, 73]. Further, it may be helpful to focus on medical conditions reported to be more prone to diagnostic errors. One target may be the three-fourths of serious misdiagnoses that are attributable to major vascular events, cancer, and infectious diseases [74, 75]. Diagnostic surveillance can provide a data-driven approach to quality management that spans both patient-care and laboratory settings may provide an approach to advancing diagnostic excellence. DMTs that include patient engagement can potentially drive this process to assure that accurate and timely diagnoses are achieved, and diagnostic errors are minimized.

Significant challenges to change include the need to prioritize efforts based on limited organizational resources, lack of effective information exchange among medical disciplines and leadership, and resistance to change and in part based on a culture that has separated laboratory from clinical care practices and processes [76]. Other challenges include laboratory regulatory and accreditation standards that, as of 2020, generally do emphasize expanded pre- and post-analytic roles of the laboratory in working with clinicians to promote accurate and timely diagnoses [56, 60,61]. Changes in practice to promote diagnostic excellence impact the business model but there is likely a value proposition for utilization management, reducing diagnostic errors and improving health care outcomes [77].

## Outlook

In crafting strategies and launching initiatives to advance diagnostic excellence and address barriers to practice changes, laboratory engagement is important. While not intended to be exhaustive, examples provided within this report suggest that laboratory practices and expertise can support a broad range of actions that lead to accurate and timely diagnoses and a reduction in diagnostic errors. Emerging technologies and communication channels provide additional opportunities for laboratories to leverage data and engage with other healthcare professionals. These include laboratory-informed decision support tools to aid clinicians in test selection and interpretation. Two advancing areas of practice worth commenting include telemedicine and point-of-care testing. Telemedicine uses technology to make remote diagnoses that include the use of various testing modalities, and is very useful for populations that do not have easy access to medical services [78]. Point-of-care testing provides the analysis where the patient is, negating the need for analysis at a distant laboratory, whether within a hospital, physician’s office, or non-medical setting [79]. While these practices do not represent new paradigms, their growing significance to healthcare delivery requires additional thought as to how these may be leveraged to promote diagnostic excellence.

With an enhanced appreciation among the practice community and patient population to improve diagnosis, it is now incumbent for healthcare professionals to continue pushing forward strategies to improve diagnoses across the medical spectrum. Integral to success for many diagnoses is engagement of clinical laboratory professionals and appreciation of the total testing process to derive accurate and timely diagnoses to inform clinician and patient decision making that ultimately contributes to improved health outcomes.

## Figures and Tables

**Table 1:** Availability of metric and performance indicators that support efforts to advance diagnostic excellence.

Quality indicators relevance	Laboratory or diagnostic setting relevance	Provided by	Primary stakeholders	Details/links/references
Prevention Inpatient Patient safety Pediatric indicators	Patient care (primarily) and laboratory	Agency for Health Research and Quality	The Public	Measures that organizations can apply to inpatient hospital data to assess and improve health care quality, identify areas that need further study and investigation, and track changes over time. (https://www.qualityindicators.ahrq.gov/Default.aspx, accessed November 4, 2020)
Adherence to clinical practice guideline recommendations Quality and performance standards Appropriate utilization of laboratory testing	Patient care and laboratory (primarily)	American Society for Clinical Pathology	Pathologists and related professionals	The National Pathology Quality Registry is a quality and benchmarking program. (https://www.ascp.org/content/get-involved/institute-of-science-technology-policy/npqr, accessed November 4, 2020)
Pre-analytic, analytic, and post-analytic process measures	Laboratory	Department of Health and Human Services (includes Centers for Medicare & Medicaid Services, the Food and Drug Administration, and the Centers for Disease Control and Prevention)	The public; regulatory activity entities providing clinical laboratory testing as a service and manufacturers of laboratory tests	Regulatory requirements for US laboratories under the clinical laboratory improvement Amendments law. (https://www.cms.gov/Regulations-and-Guidance/Legislation/CLIA, accessed November 4, 2020)
Measures for various quality, reporting, and payment programs	Patient care and laboratory	Centers for Medicare & Medicaid Services	Clinicians, clinical laboratory professionals, other healthcare professionals	Interactive web-based tool for searching through the Centers for Medicare & Medicaid Services measures inventory (https://www.cms.gov/Medicare/Quality-lnitiatives-Patient-Assessment-Instruments/QualityMeasures/CMS-Measures-lnventory, accessed November 4, 2020)
Topic-specific measures across the laboratory medicine domain	Patient care and laboratory	Clinical and Laboratory Standards Institute	Academic, commercial, and governmental entities	Many guidelines include quality indicators intended for harmonization across the international laboratory medicine community (https://clsi.org/, accessed November 4, 2020)
Laboratory benchmarks Q-Probes Q-Tracks	Patient care and laboratory (primarily)	College of American Pathologists	Clinical and anatomic pathologists	Benchmarks and quality indicators derived from short- and long-term studies that impact test results and patient outcomes. (https://www.cap.org/laboratory-improvement/quality-management-programs, accessed November 4, 2020)
Health outcome Support processes Key processes Key processes for genetic diagnoses	Patient care and laboratory (primarily)	International Federation of Clinical Chemistry And Laboratory Medicine Working Group on Laboratory Errors and Patient Safety “laboratory errors and patient safety” (IFCC WG-LEPS)	National societies of clinical chemistry and laboratory medicine; clinical laboratory industry	2017 workgroup report: Quality indicators in laboratory medicine: The status of the progress of IFCC working group “laboratory errors and patient safety” project [60]. (http://217.148.121.44/MQIWeb/Page_QualityIndicators.jsf, accessed November 4, 2020)
Accreditation and standardized performance measures for hospitals and laboratories	Patient care and laboratory	There are several federally approved accreditors. In the laboratory domain, CLIA recognizes the following accreditation organizations: AABB (formerly the American Association for Blood Banks) American Association for Laboratory Accreditation American Society for Histocompatibility and Immunogenetics American Osteopathic Association COLA (formerly the Commission on Office Laboratory Accreditation) College of American Pathologists The Joint Commission	Healthcare facilities and laboratories	Example: The Joint Commission: Nationally (US) implemented standard core performance indicators for hospitals (https://www.jointcommission.org/measurement/measures/, accessed November 4, 2020) Example: College of American Pathologists Performance standards for pathologists https://www.cap.org/advocacy/quality-payment-program-for-pathologists/mips-for-pathologists, accessed November 4, 2020)
Electronic measures designed to be implemented within health information technology systems	Patient care (primarily) and laboratory	National Quality Forum	Diverse organizations with health care focus	Primary focus is evidence-based measure endorsement. (http://www.qualityforum.org/Home.aspx, accessed November 4, 2020)

**Table 2:** Examples of federal agencies and professional groups active in addressing aspects of diagnostic excellence across patient care and clinical laboratory disciplines.

Professional organization	Primary member-ship/collaborator	Example(s) of diagnostic excellence initiatives
Agency for Healthcare Research And Quality	Federal agencies, healthcare providers, laboratory professionals, and other relevant professionals and organizations	Multifaceted efforts to address diagnostic safety and quality (https://www.ahrq.gov/topics/diagnostic-safety-and-quality.html, accessed November 4, 2020)
American Association for Clinical Chemistry	Clinical chemists, other laboratory professionals	AACC supports lab tests online, a health information web resource designed to help patients understand lab tests that contribute to making diagnoses and otherwise used in clinical practice, (https://labtestsonline.org/, accessed November 4, 2020)
American Society for Clinical Laboratory Science	Laboratory professionals (also personnel certification (majority of which are for clinical technicians and technologists)	A review of published studies on the value of laboratory medicine [62]
American Society for Clinical Pathology	Laboratory professionals (also provides certifications for laboratory professionals)	Addressing the role of the laboratory in patient safety that includes the reduction of diagnostic errors. (https://www.ascp.org/content/docs/default-source/policy-statements/ascp-pdft-pp-quality-lab-practice.pdf?sfvrsn=2, accessed November 4, 2020)
Armstrong Institute for Patient Safety and Quality, Center for Diagnostic Excellence	Healthcare professionals across disciplines and organizations	The center engages in efforts to raise awareness, measuring the impact of new diagnostic strategies, advance research, training, and infrastructure capacity to improve diagnoses. (https://www.hopkinsmedicine.org/armstrong_institute/centers/center_for_diagnostic_excellence/about.html, accessed November 4, 2020)
US Centers for Disease Control and Prevention, Division of Laboratory Systems	Federal agencies, healthcare providers, laboratory professionals, and other relevant professionals and organizations	Manages the US federal clinical laboratory quality improvement Advisory committee that has discussed and submitted recommendations to the department of health and human services regarding aspects of diagnostic excellence (https://www.cdc.gov/cliac/meeting.html, accessed November 4, 2020)
		A 2020 pilot of a laboratory community of practice (CoP) on diagnostic excellence that uses case-based studies to educate and train healthcare professionals and examine cross-cutting issues in laboratory medicine. (https://www.cdc.gov/labquality/echo.html, accessed November 4, 2020)
Choosing Wisely (under the American Board of Internal Medicine Foundation)	Laboratory professionals, clinicians, patient advocacy representatives, and subject matter experts in laboratory medicine, and other relevant stakeholders	Develops recommendations that help patients have meaningful discussions with their healthcare provider about the evidence in choosing care, that includes clinical laboratory testing, that is necessary, and free from harm, (https://www.choosingwisely.org/, accessed November 4, 2020)
Clinical Laboratory Management Association	Clinical laboratory professionals, managers, and leaders	Course: The impact of laboratory services on diagnostic errors (https://www.clma.org/p/bl/et/blogid=64&blogaid=388, accessed November 4, 2020)
International Federation of Clinical Chemistry and Laboratory Medicine Working Group on Laboratory Errors and Patient Safety (IFCC WG-LEPS)	National societies of clinical chemistry and laboratory medicine; diagnostic companies	2017 workgroup report: Quality indicators in laboratory Medicine: the status of the progress of IFCC Working Group “Laboratory Errors and Patient Safety” project. [60]
The Joint Commission	Healthcare facilities and laboratories (also provides accreditation and certification)	Safety Advisory: Reducing diagnostic error through closed loop communication (https://www.jointcommission.org/resources/news-and-multimedia/news/2019/12/the-joint-commission-issues-quick-safety-advisory-on-reducing-diagnostic-errors/, accessed November 4, 2020)
Clinical Lab 2.0, a project initiative by Santa Fe Foundation Initiative	Clinical laboratories, clinical laboratory professionals, and businesses that support clinical laboratory practice	Promotes development of the evidence base for the valuation of clinical laboratory services in the next era of global healthcare that includes the optimization of diagnostic processes. [63] (https://www.cl2lab.org/, accessed November 4, 2020)
Society to Improve Diagnosis in Medicine	Health care and clinical laboratory professionals	Broad focus dedicated to improving diagnosis that includes the laboratory component (https://www.improvediagnosis.org/, accessed November 4, 2020)
US Department of Veterans Affairs, Health Policy, Quality & Informatics Program	Federal agencies, healthcare providers, laboratory professionals, and other relevant professionals and organizations	Engages in multidisciplinary research and uses findings to reform clinical practice, redesign care processes and inform policy development to improve quality, safety, and effectiveness of healthcare, that includes a focus on diagnostic excellence. (https://www.houston.hsrd.research.va.gov/health-policy/hpqi.asp, accessed November 4, 2020)
